# Correction: Investigations of the mechanisms of interactions between four non-conventional species with *Saccharomyces cerevisiae* in oenological conditions

**DOI:** 10.1371/journal.pone.0253680

**Published:** 2021-06-17

**Authors:** Olivier Harlé, Judith Legrand, Catherine Tesnière, Martine Pradal, Jean-Roch Mouret, Thibault Nidelet

The first author’s name is spelled incorrectly. The correct name is: Olivier Harlé.
