# Supplementary figures and images for: Investigations of the mechanisms of interactions between four non-conventional species with Saccharomyces cerevisiae in oenological conditions
Source: PLoS One. 2020 May 26;15(5):e0233285. doi: 10.1371/journal.pone.0233285 (PMC7250438; doi:10.1371/journal.pone.0233285)

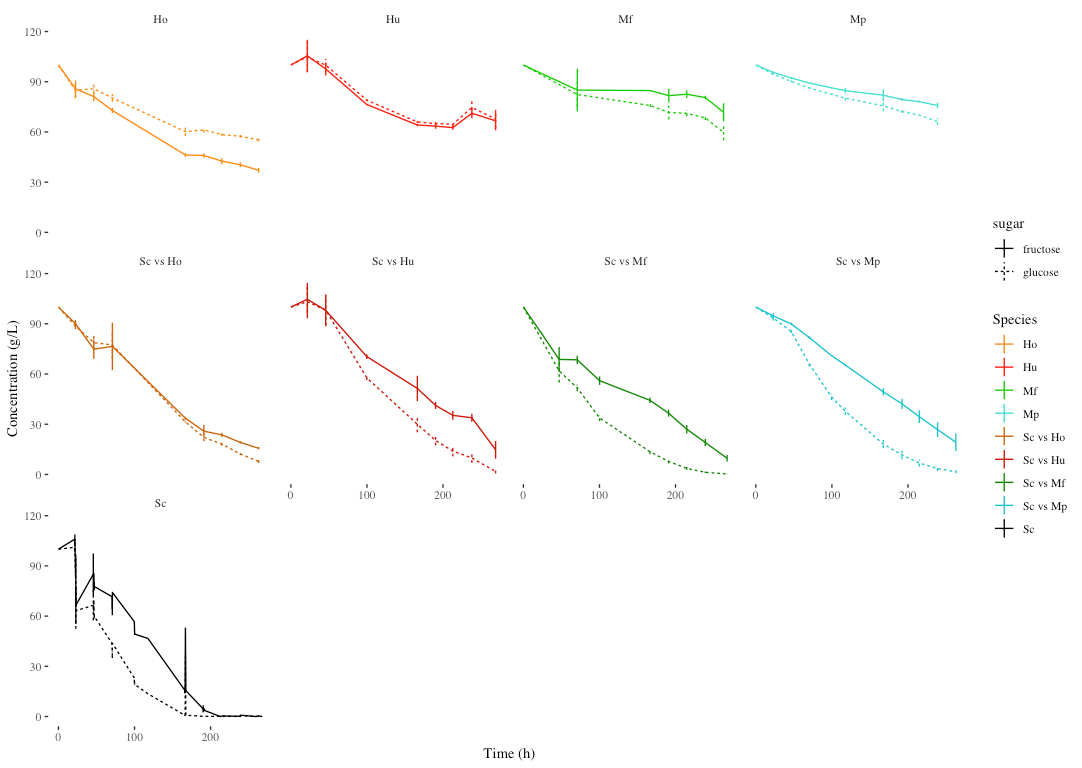

Supplement: S1 Fig — Each point represents a sample (average ± standard deviation). (TIF) [file pone.0233285.s001.tif]

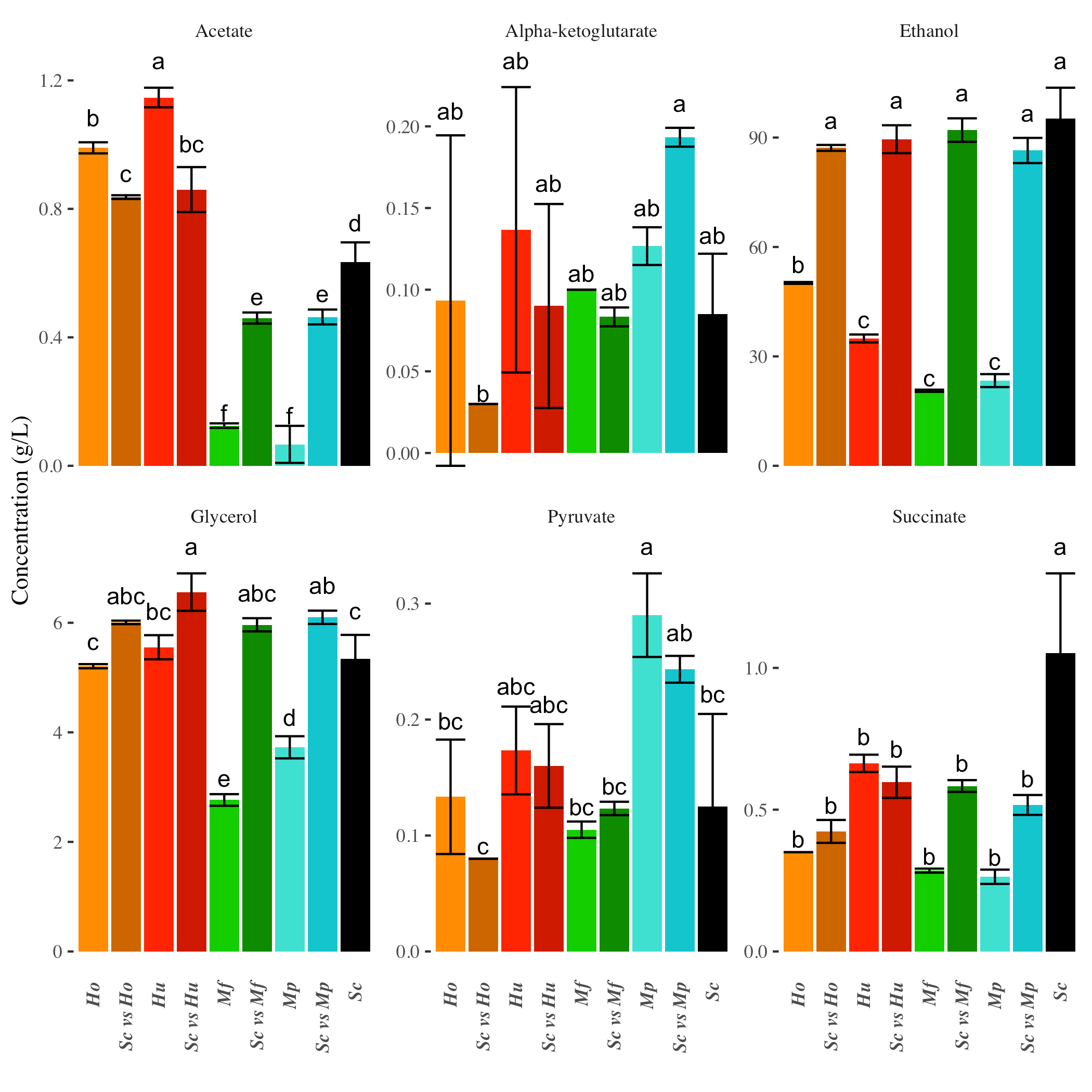

Supplement: S2 Fig — Average production are given with standard deviations for acetate, alpha-ketoglutarate, ethanol, glycerol, pyruvate and succinate. (TIF) [file pone.0233285.s002.tif]

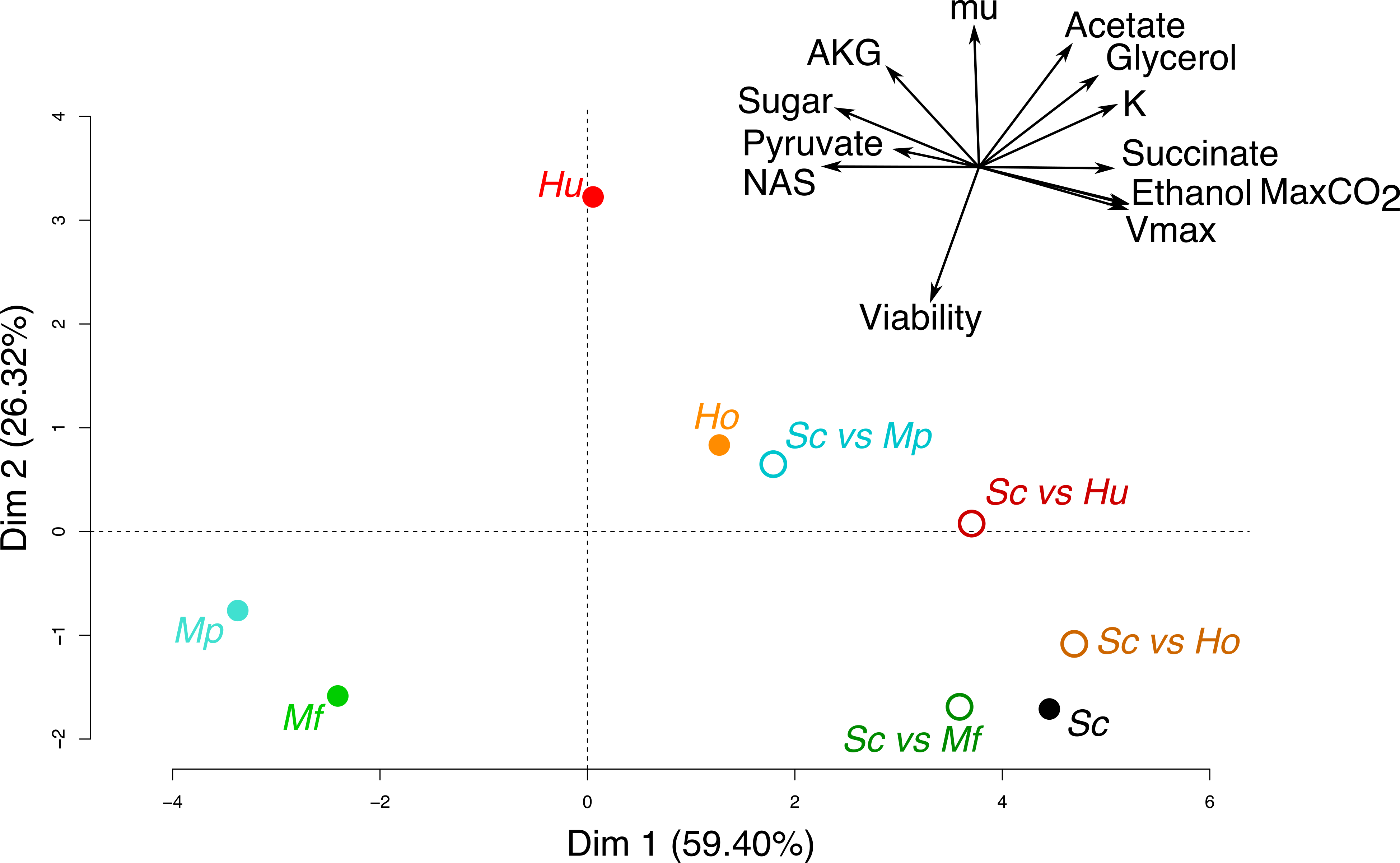

Supplement: S3 Fig — The mixed cultures are a second time projected on the plan determiner by only monocultures. In the top right is represented the circle of variables. (TIF) [file pone.0233285.s003.tif]

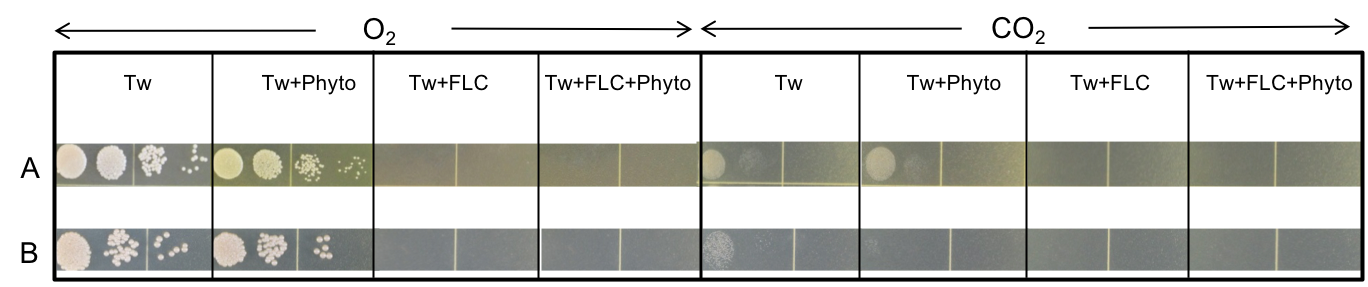

Supplement: S4 Fig — Serial tenfold dilutions of two Metschnikovia pulcherrima strains (A and B) spotted onto various synthetic standard agar media (SM425, 425 mg/l assimilable nitrogen) with Tween 80, (Tw, 0.06%), supplemented or not with phytosterol (Phyto, 20 mg/L), in the presence or not of fluconazole (FLC, 256 μg/mL). Plates were incubated at 28°C for five days in air or in anaerobiosis (TIF) [file pone.0233285.s004.tif]
