# Supplementary material for: Investigations of the mechanisms of interactions between four non-conventional species with Saccharomyces cerevisiae in oenological conditions
Source: PLoS One. 2020 May 26;15(5):e0233285. doi: 10.1371/journal.pone.0233285 (PMC7250438; doi:10.1371/journal.pone.0233285)
Supplement: S1 Table — (DOCX) [file pone.0233285.s005.docx]

**S1 TABLE**

**Growth parameter values for each type of fermentation.**

***µ*** K prop Vmax MaxCO2 Sugar NAS Ethanol Glycerol Succinate Acetate Pyruvate Alpha

***Sc*** 0.15±0.02**^bc^** 1.6±0.2**^bcd^** NA 0.99±0.03**^a^** 88.2±2.2**^a^** 0.2±0.2**^d^** 10.4±8.6**^c^** 95.2±8.4**^a^** 5.3±0.4**^c^** 1.05±0.28**^a^**  0.63±0.06**^d^**  0.12±0.08**^bc^** 0.08±0.04**^ab^**

***Ho*** 0.19±0.03**^bc^** 1.6±0.1**^bc^** NA 0.42±0.02**^d^** 46.4±0.6**^d^** 92.3±1.4**^b^** 122.2±3.5**^b^** 50.1±0.2**^b^** 5.2±0.0**^c^** 0.35±0.00**^b^** 0.99±0.02**^b^** 0.13±0.05**^bc^** 0.09±0.1**^ab^**

***ScvsHo*** **0.11±0.00^bc^** **2.3±0.1^a^** 0.83±0.02**^b^** 0.57±0.01**^c^** 82.7±0.6**^bc^** 23.3±1.3**^c^  6.4±2.4^c^** 87.2±0.8**^a^** **6.0±0.0^abc^** 0.42±0.04**^b^**  0.84±0.01**^c^** **0.08±0.00^c^** **0.03±0.0^b^**

***Hu*** 0.62±0.18**^a^** 1.4±0.1**^cde^** NA 0.34±0.04**^d^** 30.7±0.5**^e^** 134.3±11.2**^a^** 173.3±24.8**^b^** 34.9±1.1**^c^** 5.5±0.2**^bc^** 0.66±0.03**^b^**  1.15±0.03**^a^** 0.17±0.04**^abc^** 0.14±0.09**^ab^**

***ScvsHu*** 0.24±0.01**^b^** **1.7±0.1^b^** 0.76±0.01**^c^** 0.53±0.04**^c^** 82.1±1.5**^c^** 16.5±6.5**^c^** **4.6±4.2^c^** 89.5±3.8**^a^**  **6.6±0.3^a^** **0.60±0.06^b^** 0.86±0.07**^bc^** 0.16±0.04**^abc^** 0.09±0.06**^ab^**

***Mf*** 0.17±0.02**^bc^** 0.8±0.05**^f^** NA 0.17±0.04**^e^** 20.6±1.2**^f^** 135.8±9.7**^a^** 256.6±2.9**^a^** 20.6±0.3**^c^** 2.8±0.1**^e^** 0.29±0.01**^b^** 0.13±0.01**^f^** 0.10±0.01**^bc^** 0.10±0.00**^ab^**

***ScvsMf*** **0.09±0.01^bc^** 1.3±0.03**^de^** 0.96±0.02**^a^** 0.76±0.03**^b^** 86.8±0.7**^ab^** 9.9±2.1**^cd^**  **1.8±0.1^c^** 92.1±3.2**^a^**  **6.0±0.1^abc^** 0.58±0.02**^b^**  0.46±0.02**^e^** 0.12±0.01**^bc^** 0.08±0.01**^ab^**

***Mp*** 0.18±0.03**^bc^** 0.6±0.01**^f^** NA 0.16±0.02**^e^** 21.8±0.4**^f^** 142±3.7**^a^** 305.5±5.9**^a^** 23.4±1.8**^c^** 3.7±0.2**^d^** 0.26±0.03**^b^** 0.07±0.06**^f^** 0.29±0.04**^a^** 0.13±0.01**^ab^**

***ScvsMp*** **0.09±0.00^c^** 1.3±0.01**^e^** 0.50±0.02**^d^** 0.68±0.04**^b^** 83.3±2.2**^bc^** 20.8±6.0**^c^** **2.1±0.2^c^** 86.5±3.4**^a^** **6.1±0.1^ab^** 0.52±0.04**^b^** 0.46±0.02**^e^** 0.24±0.01**^ab^** **0.19±0.01^a^**

Mu : h-1; K : 10^8^ cells.mL-1, prop : no unity, Vmax : g.L-1.h-1, MaxCO2 : g.L-1, Final Sugar : g.L-1, Final NAS : mg.L-1, Ethanol : g.L-1, Glycerol : g.L-1, Succinate : g.L-1, Acetate : g.L-1, Pyruvate : g.L-1, Alpha : g.L-1

**Bold:** values of mixed culture that are not intermediate between the two values of the corresponding monocultures, a sign of transgressive interactions.

Superscript: Statistical groups obtained from a Tukey test.
