# Supplementary material for: Investigations of the mechanisms of interactions between four non-conventional species with Saccharomyces cerevisiae in oenological conditions
Source: PLoS One. 2020 May 26;15(5):e0233285. doi: 10.1371/journal.pone.0233285 (PMC7250438; doi:10.1371/journal.pone.0233285)
Supplement: Attachment — Submitted filename: renamed_8b108.docx [file pone.0233285.s006.docx]

Reviewer #1: The authors made the following assumption : « all cultures that produced more than 80g CO2.L-1 (90% of Sc maximum) within 300 hours will be able to complete fermentation »

This assumption is not supported by any scientific evidence. The authors should have followed the entire fermentation kinetics in order to interpret the data correctly.

This part was not clearly described, thus the sentence has been completely rewrote to clarify the point and data concerning the maximum level of CO2 produced have been given. See lines 125-130. Moreover, this point has been discussed in the discussion section (see lines 251-264 with reference added).

In the summary the authors stated : » M. pulcherrima and M. fructicola displayed a negative interaction with the S. cerevisiae strain tested, with a decrease in viability in co-culture, probably due to iron depletion via the production of pulcherriminic acid.

The authors suggest iron depletion to be responsible for viability decrease, however, no results presented in this paper support this hypothesis, already reported.

Summary is not the place for such assumption.

The sentence “probably due to iron depletion via the production of pulcherriminic acid” has been removed from the abstract (see lines 35-36).

The first part of the discussion is redundant with the result.

This part has been rewrote to take this remark into account (see lines 244-365 with reference added).

One of the conclusion is : Meanwhile NS fermentations present similarities, it was possible to group the NS strains performance according to their genus.

This is an overinterpretation, taking into account the small number of tested genus and tested strains. This point has been discussed lines 257-270.

The overall discussion is quite poor with very few references to support the comments. Numerous changes have been performed in particular with the addition of new references to support our comments (lines 244-365).

For example, regarding the role of lipids that could play a role in the interactions, there is no reference, while some works do exist. References have been added (see paragraph 285-302).

CO2 should be written CO2 in legend (done)

Reviewer #2: The manuscript is concise and easy to follow. The authors have evaluated the interactions between Saccharomyces cerevisiae and four non-Saccharomyces species with focus on metabolic interactions in particular nutrient resource utilization. A couple of different interaction mechanisms could be identified. One limitation to this study is that only single strains were used for each species, and the authors should at least acknowledge the fact that there might be strain variability and briefly discuss this. Overall, the study set-up is sound but would have benefited from multiple strains per species.

Some minor corrections in text are necessary to improve the quality of the paper. These are as follows:

Throughout the document, replace “isolated cultures” with either ‘single cultures’ or ‘monocultures’ “Isolated cultures” has been replaced by “Monocultures” lines 94, 161 and throughout the paper.

The use of “strain” in this manuscript is confusing since only single strains were used for each species. Perhaps the authors should rather consider just referring to the species. This has been done throughout the paper.

All the images used for the figures are poor quality and no easy to read through

Page 2 line 8: “and” should not be in italics. (This has been done line 28.)

Page 3 line 6: “aiming” should be aimed (done line 64)

Page 4 line 75: “smocky” should be smoky (done line 74)

Page 5 line 101: “choose” should be chose (done line 100)

Page 6 line 135: “corresponds” should be correspond (done line 134)

Page 7 line 147: change “on the opposite” to Conversely or In contrast (done lines 147)

Page 7 line 151: “µm” should be µM (done line 151)

Page 8 line 178: insert space between 300 and h (done line 179)

Page 8 line 185-186: the part of the sentence starting with “whereas” does not make sense (sentence changed lines 187-188)

Page 9 line 217: change “isolated” to monoculture (done line 210)

Page 9-10 line 217-219: the author indicates no significant difference between mixed cultures and their corresponding mono cultures, but this is not true for the Hu and Mp cultures. (This has been clarified lines 218-220)

Page 11 line 252: “decreasing” is in a different font type from the rest of the text OK

Page 12 line 275: which PCA and Figure 5 is the author referring to? The paragraph has been removed.

Page 12 line 280: remove “nevertheless” and start the sentence with “Despite” The paragraph has been removed.

Page 12 line 286 – 290: the authors should refer to studies that have been done on oxygen e.g. Shekhawat et al. 2017 and 2018, Morales et al., 2015 (references added line 297)

Page 14 line 320: replace “evidence” with observe (OK)
